# The impact of ruminative thought style on the maintenance of depressive mood

**DOI:** 10.1192/j.eurpsy.2024.544

**Published:** 2024-08-27

**Authors:** V. Misic, T. Vukosavljevic Gvozden, B. Batinic

**Affiliations:** ^1^Department of Psychology, University of Belgrade, Faculty of Philosophy; ^2^University Clinical Centre of Serbia, Clinic of Psychiatry, University of Belgrade, Faculty of Philosophy, Department of Psychology, Belgrade, Serbia

## Abstract

**Introduction:**

Ruminations are a cognitive style of “thought recycling”, which involves passively and repeatedly focusing on disorder and distress symptoms, or their causes, without attempting to alleviate them. They are a significant indicator of cognitive vulnerability, predicting the emergence, maintenance, and recurrence of depressive symptoms.

**Objectives:**

To estimate the impact of the ruminative thought style on the maintenance and escalation of depressive mood.

**Methods:**

The research sample consisted of 60 students between the ages of 19 and 30 (M = 23), divided into two experimental groups with 30 participants each. The participants took part in a 5-minute experiment that involved recalling an autobiographically sad event, assessing their mood on the Scale for Self-Assessment of Emotions (EAS) before and after the induction, and then splitting into two groups of 30 participants for random ruminating or distraction. The Beck Depression Inventory-II, the Ruminative Response Scale, the Ruminative Thought Style Questionnaire, and the EAS were used as research instruments. The progressive group relaxation approach was used at the end of the experiment with all participants to promote relaxation and lessen the psychophysical tension brought on by the experimental induction (10 minutes total).

**Results:**

The experimental groups did not differ in mood intensity prior to the induction of sadness. Both experimental groups experienced significant impacts on depressed mood following the induction of sadness (F (1,58) =92.05, p<0.001): participants who ruminated demonstrated persistence in their negative mood, whereas participants who engaged in distractions demonstrated a decrease in their negative mood, even below the initial level (F (2,116) =12.69, p<0.001).

**Conclusions:**

This result provides an additional experimental validation of the phenomenon of maintaining a depressive mood through ruminations. An essential psychotherapy goal should be the treatment (metacognitive therapy, rumination-focused CBT, mindfulness, cognitive bias correction, etc.) of such mechanisms, recognized as crucial for the maintenance of depression.

**Disclosure of Interest:**

None Declared

